# Comparative Study of Thermal and Plasma-Enhanced Atomic Layer Deposition of Iron Oxide Using Bis(*N,N*′-di-butylacetamidinato)iron(II)

**DOI:** 10.3390/nano13121858

**Published:** 2023-06-14

**Authors:** Boyun Choi, Gun-Woo Park, Jong-Ryul Jeong, Nari Jeon

**Affiliations:** Department of Materials Science and Engineering, Chungnam National University, Daejeon 34134, Republic of Korea; bow06123@o.cnu.ac.kr (B.C.); adgv9073@gmail.com (G.-W.P.); jrjeong@cnu.ac.kr (J.-R.J.)

**Keywords:** thermal atomic layer deposition, plasma-enhanced atomic layer deposition, bis(*N,N*′-di-butylacetamidinato)iron(II) (FeAMD), iron oxides, hematite, high aspect ratio, trench structures

## Abstract

Only a few iron precursors that can be used in the atomic layer deposition (ALD) of iron oxides have been examined thus far. This study aimed to compare the various properties of FeO_x_ thin films deposited using thermal ALD and plasma-enhanced ALD (PEALD) and to evaluate the advantages and disadvantages of using bis(*N,N*′-di-butylacetamidinato)iron(II) as an Fe precursor in FeO_x_ ALD. The PEALD of FeO_x_ films using iron bisamidinate has not yet been reported. Compared with thermal ALD films, PEALD films exhibited improved properties in terms of surface roughness, film density, and crystallinity after they were annealed in air at 500 °C. The annealed films, which had thicknesses exceeding ~ 9 nm, exhibited hematite crystal structures. Additionally, the conformality of the ALD-grown films was examined using trench-structured wafers with different aspect ratios.

## 1. Introduction

Hematite (α-Fe_2_O_3_) films can be used in many devices, including water splitters [1,2,3,4,5,6,7,8,9,10,11], gas sensors [12,13,14,15,16,17], and supercapacitors [18,19,20,21], in different fields. Therefore, various methods of preparing hematite films, such as sputtering [4,5,6,22], molecular beam epitaxy [23,24,25,26], chemical vapor deposition (CVD) [14,16,27,28,29], sol-gel method [15,30,31], hydrothermal synthesis [1,32], and spray pyrolysis [7,12,18,21], have been explored. Atomic layer deposition (ALD) has unique advantages over other deposition techniques because of its ability to accurately control the film thickness at the angstrom level and ensure a uniform film thickness on the wafer scale along with conformal film deposition on three-dimensional nanostructures with complex geometries. Several studies have investigated the ALD of iron oxides (FeO_x_) using different ALD precursors, such as ferrocene [11,33,34], t-butylferrocene [35,36], *N*,*N*-(dimethylaminomethyl)ferrocene [37], tris(2,2,6,6-tetramethyl-3,5-heptanedionato)iron(III) (Fe(thd)_3_) [38,39], and FeCl_3_ [40]. The detailed conditions used in ALD and resultant properties of the films deposited largely depend on the surface reactions between the ALD precursors and terminal functional groups. Therefore, studies on the use of new types of precursors in ALD are consistently encouraged in the field of ALD.

The objective of this study was to evaluate the viability of using bis(*N,N*′-di-butylacetamidinato)iron(II) (FeAMD) as an iron precursor in the preparation of FeO_x_ thin films. To this end, the properties of FeO_x_ thin films deposited using thermal ALD and plasma-enhanced ALD (PEALD) with FeAMD as the precursor were compared. Avila et al. first reported the thermal ALD of FeO_x_ thin films using FeAMD [41]; however, no further research has been reported on the use of FeAMD in FeO_x_ ALD. The novelty of this study is that it dealt with the practical aspects of using FeAMD as an ALD precursor by considering the vapor pressure during deposition along with the thermal stability and chemical/physical properties of the deposited films. Moreover, this paper is the first to report a FeO_x_ PEALD process using FeAMD. In this study, both thermal ALD and PEALD films were comprehensively characterized using spectroscopic ellipsometry (SE), atomic force microscopy (AFM), grazing incidence X-ray diffraction (GIXRD), X-ray photoelectron spectroscopy (XPS), and scanning electron microscopy (SEM).

## 2. Materials and Methods

A PEALD reactor system with an 8-inch wafer chuck was used for both thermal ALD and PEALD processes. The PEALD reactor included an inductively coupled plasma system with a remote plasma geometry; the plasma shower head was located 89 mm away from the sample plate. The reactor had a cross-flow design in which the precursor vapors (FeAMD or H_2_O) flowed across the substrate surface; the precursor delivery line was separated from the plasma gas line. For the ALD process, FeAMD (product number: 26-0145, Strem Chemicals, Inc., Newburyport, MA, USA) was used as the Fe precursor, and either H_2_O or O_2_ plasma was used as the oxidizing coreactant. The temperatures of the FeAMD canister, gas line, and substrate were 150, 200, and 200 °C, respectively. During the entire ALD process, Ar carrier gas at 5 SCCM was continuously provided along the two gas lines delivering the precursor (FeAMD or H_2_O) vapor and plasma gas. During the oxygen plasma step, oxygen was provided along with Ar carrier gas at an increased flow rate (50 SCCM). The duration of a single dose of FeAMD was maintained at 1 s. The dose durations of H_2_O (0.1–2 s) and oxygen plasma (5–120 s) were varied as described in Section 3. In the FeAMD half cycles, a purge time of 300 s was chosen to allow sufficient time for FeAMD precursor evaporation. A Si wafer (p type, 1–10 Ωcm) with an approximately 2 nm native oxide layer was used as the substrate after being cleaned via sonication with ethanol and isopropanol.

The film thicknesses were measured at four wavelengths, namely 463, 523, 600, and 637 nm, using SE (FS-1, Film Sense LLC, Lincoln, NE, USA). The SE data were fitted using three parameters: refractive index, extinction coefficient, and film thickness. AFM (XE7, Park Systems Corp., Suwon, Republic of Korea) was used to measure the surface roughness of the films in the contact mode with a scan size of 5 μm × 5 μm and at a scan rate of 1 Hz. XPS (K-alpha, Thermo Fisher Scientific Inc., Waltham, MA, USA) with an Al K-α source of 1 keV was used to determine the chemical composition of the films. The XPS data were collected after 5 s of etching to enable surface contamination. The cross-sectional images of the films deposited on the trench-structured substrate were analyzed using SEM (Verios 5 UC, Thermo Fisher Scientific Inc., Waltham, MA, USA). An X-ray diffractometer (SmartLab, RIGAKU Co., Ltd., Tokyo, Japan) was used to collect the GIXRD data in the two-theta scan mode at a scan speed of 1.5°/min and with a step size of 0.02°.

## 3. Results and Discussion

A conventional ALD cycle consists of two half cycles with each cycle comprising a metal precursor (or coreactant) dosing step followed by a metal precursor (or coreactant) purging step. Multiple doses of the precursor may be administered within a single half cycle to ensure the saturation of the surface reactions between the surface functional groups and precursor, particularly when the vapor pressure of the precursor is insufficient. In this study, the number of mini doses within a single-half cycle of FeAMD was varied from one to five, while the oxidant half cycle consisted of a single oxidant dose. We employed the multiple dose scheme to evaluate the necessary FeAMD exposure amount for achieving the saturation of precursor surface coverage within a half cycle, as discussed in this work (*vide infra*).

**Figure 1:**
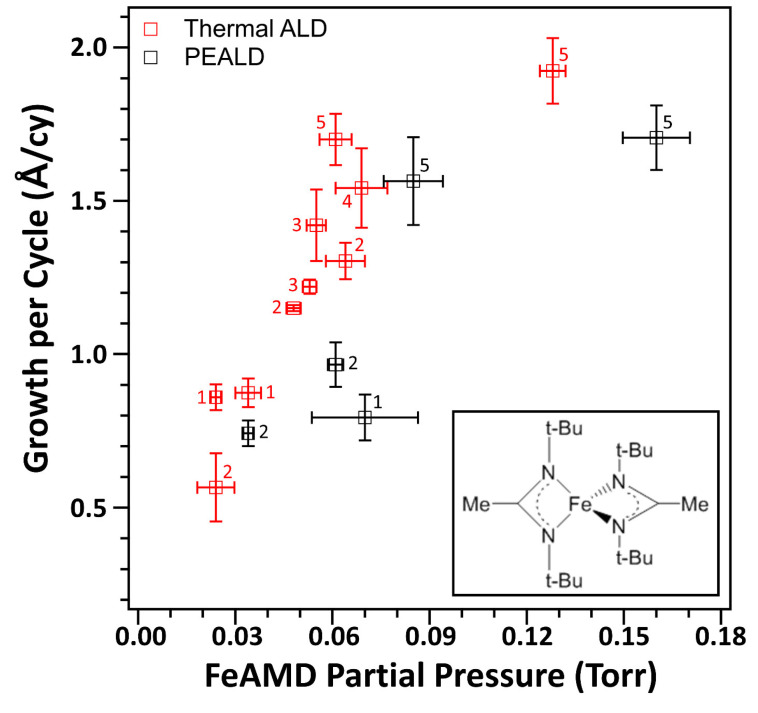
Growth per cycle of FeO_x_ thin films deposited using thermal atomic layer deposition (ALD) and plasma-enhanced ALD (PEALD) at different bis(*N,N*′-di-butylacetamidinato)iron(II) (FeAMD) partial pressures. During PEALD, oxygen plasma power and dose time were kept at 20 W and 60 s, respectively. The inset shows the chemical structure of FeAMD. The number of mini doses in each ALD experiment was indicated in the figure.

Figure 1 shows the growth per cycle (GPC) of the FeO_x_ film grown using 100 ALD cycles, as a function of the average partial pressure of FeAMD during a “half” cycle (not with a mini dose). An example of a partial pressure measurement is shown in Figure S1. The vertical error bars in the GPC plot were measured using five samples located on the 8-inch wafer chuck at a considerable distance from one another. The horizontal error bars related to the partial pressure were calculated using ten representative ALD cycles under thermal ALD and PEALD conditions. The GPC values under both conditions tended to increase with increasing partial pressure until they reached approximately 80 mTorr. The saturated GPC values were within the range of 1.7–1.9 Å/cy at high partial pressures (>100 mTorr). We obtained the high partial pressure when using five mini doses of FeAMD, indicating that five mini doses are suggested as the optimal number. The GPC values measured in this study were greater than those measured using FeO_x_ ALD processes that used other Fe precursors, such as Fe(thd)_3_, (0.14 Å/cy) [38], *N,N*-(dimethylaminomethyl)ferrocene (0.26 Å/cy) [37], and ferrocene (1.4 Å/cy) [33]. The GPC values were three to four times higher than those reported in a previous paper regarding the same precursor (FeAMD) [41]. This discrepancy could be attributed to the low thermal stability of the FeAMD precursor. The decomposition of FeAMD started at approximately 160 °C [42]. In this study, FeAMD was heated to 150 °C to obtain a sufficient vapor pressure while minimizing precursor decomposition. Even with a long purge (300 s) between two subsequent mini doses, the FeAMD partial pressure tended to decrease gradually during the ALD runs, probably due to the agglomeration of FeAMD powders subsequent to prolonged heating. To maintain a constant vapor pressure, the agglomerated FeAMD powders had to be broken down into small pieces two or three times during the 100 ALD cycles (Figure S2). The GPC values of the PEALD films were lower than those of the thermal ALD films owing to the higher density of the PEALD films (approximately 4.9 g/cm^3^) compared with those of the thermal ALD films (approximately 4.0 g/cm^3^) measured using X-ray reflection spectroscopy (Figure S3). Furthermore, the saturation behaviors of FeO_x_ films at different oxidant doses (H_2_O and oxygen plasma) were confirmed (Figure S4). ALD experiments were also performed with only the FeAMD half cycle (FeAMD partial pressure: ~96 mTorr)—that is, without the coreactant half cycle—to study the potential contribution of the thermal decomposition of FeAMD to the growth of FeO_x_ films. The grown film had a GPC of approximately 1 Å/cy, which was significantly lower than that of either thermal ALD or PEALD films (approximately 1.6–1.7 Å/cy) at the similar FeAMD partial pressures. These results suggest that the contribution made by the thermal decomposition of FeAMD to the film deposition cannot be disregarded. An ALD-like component in GPC measures approximately 0.6–0.7 Å/cy, which closely aligns with the GPC value observed by Avila et al. [41]. The presence of the non-negligible CVD component is a major disadvantage of FeAMD because well-saturated GPC curves have been reported when using FeO_x_ ALD with FeCl_3_ [40]. Future research directions could include the rational design of the molecular structures of Fe precursors to optimize their thermal stabilities and chemical reactivities toward oxidizing coreactants [43]. Additionally, technological developments in the design of precursor canisters and gas delivery systems may facilitate the efficient delivery of FeAMD vapors with reduced possibilities of FeAMD decomposition [44]. The GPC graph (Figure 1) may be drawn as a function of the number of mini doses. However, the partial pressure can be a more representative parameter of the number of FeAMD molecules provided in each FeAMD half cycle than the number of mini doses because of the problems associated with powder agglomeration.

**Figure 2:**
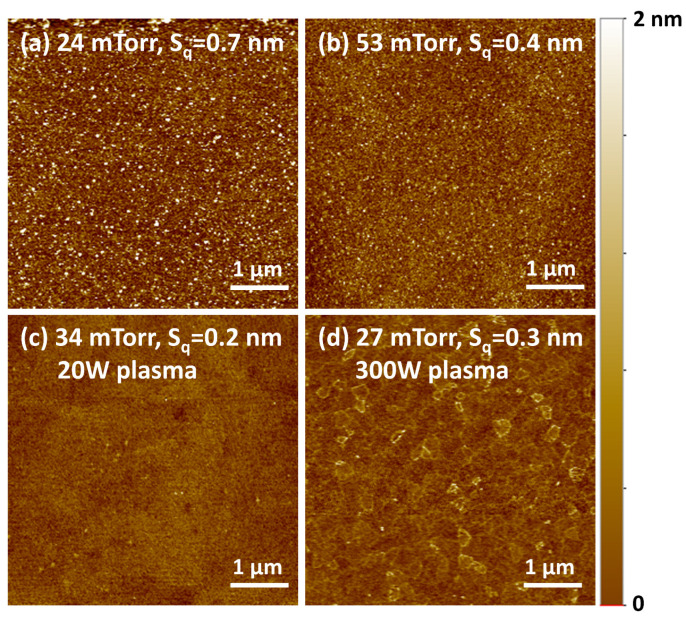
(**a**,**b**) Atomic force microscopy (AFM) images of the films obtained using thermal atomic layer deposition (ALD) and (**c**,**d**) AFM images of the plasma-enhanced ALD films grown under different conditions as indicated in the figures and the measured values of surface roughness (S_q_).

Figure 2 shows the AFM images of the FeO_x_ films grown using thermal ALD at different FeAMD partial pressures and using PEALD at different FeAMD partial pressures and oxygen plasma powers. The roughness values of the films were in the range of 0.2–0.7 nm. During the thermal ALD process, the surface roughness slightly improved as the FeAMD partial pressure increased from ~24 to 53 mTorr, possibly because of the high surface coverage of the precursors at high partial pressures. The PEALD films exhibited a slightly lower surface roughness than the thermal ALD films grown at the similar FeAMD partial pressures. The difference between the surface roughness values of the two PEALD films prepared using 20 and 300 W oxygen plasmas was negligible. Overall, both thermal ALD and PEALD films with thicknesses < 12 nm exhibited excellent surface roughness on the subnanometer scale. The variation in the surface roughness with film thickness is discussed later in the paper.

High-resolution XPS (HRXPS) was performed to determine the chemical compositions of the films (Table 1). A marginal difference existed between the chemical compositions of the two thermal ALD films grown using different H_2_O dose durations (0.1 and 2 s). Both films contained C (<7 at.%) and N (~1 at.%) impurities and were slightly substoichiometric in nature (Fe_2_O_2.5–2.6_). The Si components originated from the Si substrates because the films deposited were thin (~9–20 nm). The PEALD film had a chemical composition (~6 at.% C and ~1 at.% N impurities) and an Fe–O stoichiometric nature (Fe_2_O_2.6_), similar to the thermal ALD films. The films deposited using only FeAMD half cycles (hereby denoted as “CVD-like film”) contained higher concentrations of C (~14.5 at.%) and N (1.7 at.%) impurities than the thermal ALD and PEALD films.

**Figure 3:**
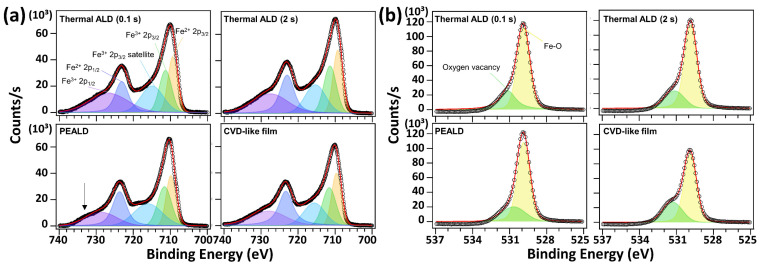
(**a**) Fe 2p and (**b**) O 1s high-resolution X-ray photoelectron spectra (HRXPS) of the thermal atomic layer deposition (ALD), plasma-enhanced ALD, and chemical vapor deposition (CVD)-like films deposited under the different conditions indicated in each figure. The baselines of the HRXPS were subtracted using the Shirley model. (The symbols in the figure indicate as follows; black circles: raw data and red lines: fitted data).

The Fe 2p HRXPS spectra of the four samples (Figure 3a) were deconvoluted into five peaks, namely an Fe^2+^ 2p_3/2_ peak at ~709.9 eV, Fe^3+^ 2p_3/2_ peak at ~711.6 eV, Fe^3+^ 2p_3/2_ satellite at ~716.0 eV, Fe^2+^ 2p_1/2_ peak at ~723.7 eV, and Fe^3+^ 2p_1/2_ peak at ~728.6 eV. The four films contained a mixture of Fe^2+^ and Fe^3+^ components, and this composition agrees with the substoichiometric nature of the films. The component within the PEALD film that exhibited the highest binding energy (BE), marked by a black arrow, was positioned approximately 1.6 eV higher in BE, and this component displayed a broader full width at half maximum. This suggests the presence of an Fe^3+^ 2p_1/2_ satellite peak and indicates an increased contribution from the Fe^3+^ component. The O 1s HRXPS spectra (Figure 3b) of the four films indicated an Fe–O component at approximately 529.9 eV with a small shoulder corresponding to the O vacancy component at ~531.1 eV. The CVD-like films exhibited a higher proportion of O vacancies than the Fe–O components. The N 1s HRXPS spectra of the CVD-like films showed a higher intensity of Fe–N than the N 1s HRXPS spectra of the other samples (Figure S5). Thus, the deposited FeO_x_ films would be of poor quality because of the limited thermal stability of the FeAMD precursors. After the completion of the ALD process, the films deposited via a CVD-like reaction (composed mainly of surface-sorbed FeAMD molecules) were oxidized to form FeO_x_ upon exposure to the O_2_ in air.

**Figure 4:**
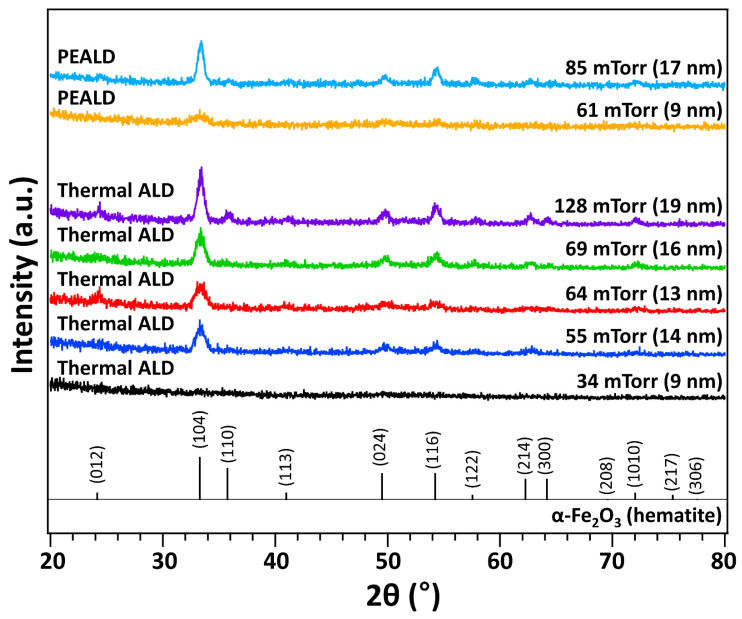
Grazing incidence X-ray diffraction patterns of the FeO_x_ thin films deposited using thermal atomic layer deposition (ALD) and plasma-enhanced ALD. Peak assignment was performed using the reference ICDD card No. 00-001-1053, which is shown at the bottom of the graph. The film thickness and partial pressure of FeAMD used for the deposition are indicated.

Figure 4 compares the crystallinities of the films grown using thermal ALD and PEALD under different conditions and obtained using the GIXRD measurements of the samples. All samples were annealed in air at 500 °C for 2 h before taking the GIXRD measurements. The hematite phase was identified in most of the annealed samples. The films were grown using a fixed number of ALD cycles (100 cycles) and different FeAMD partial pressures (film thicknesses). A majority of the thermal ALD and PEALD films exhibited a polycrystalline nature, and their crystallinity increased with increasing film thickness. In addition, for the similar thicknesses and FeAMD partial pressures, the PEALD films exhibited better crystallinity than thermal ALD films. The formation of an amorphous phase in the as-deposited films is consistent with the results of previous studies on FeO_x_ ALD using other precursors [33,41]. Crystalline phases were found in the films in as-deposited states only when the deposition temperature (>350 °C) [34] or the film thickness (>35 nm) [39,40] was significantly higher.

**Figure 5:**
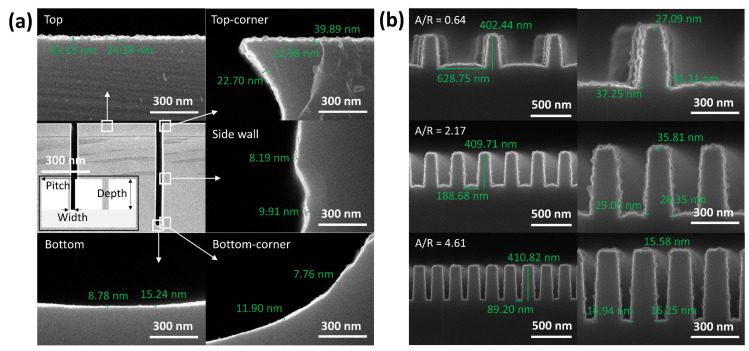
Cross-sectional SEM images of the FeO_x_ thin films deposited on patterned wafers with (**a**) high AR and (**b**) low AR. The inset of Figure 5a shows the schematic of a patterned wafer with a trench (width: 5.0 µm, depth: 65.0 µm, and pitch: 52.5 µm). The images on the left side of Figure 5b show the pattern height and space width of the patterned wafers with different ARs.

Finally, thermal ALD was performed using two different types of wafers with trench structures, namely (1) a high aspect ratio (AR) trench structure (AR = 1:13) with a 65.0 μm trench depth (Figure 5a) and (2) low AR trench structures (AR = ~0.6, 2.2, and 4.6) with a trench depth of ~400 nm (Figure 5b). The step coverage of the high AR trench structure was ~ 49%, indicating the necessity for prolonged precursor exposure. The film deposited at the bottom of the trench structure was smoother than that deposited at the top, suggesting that the surface roughness had increased as film deposition continued. The surfaces of the thin films deposited using either thermal ALD or PEALD were significantly smooth (Figure 2). The accurate measurement of the step coverage of the low AR structures was difficult because of their high surface roughness values. However, the rough surface morphology observed at the bottom of the structures implied that film growth was not limited to deep trench structures with low ARs.

## 4. Conclusions

The properties of thermal and PEALD films were examined using various characterization methods, including SE, XPS, AFM, XRD, and SEM. The GPC values of the PEALD films were ~0.2 Å/cy lower than those of the thermal ALD films because of the higher density of the PEALD films. The thermal and PEALD films contained carbon and nitrogen impurities with similar atomic concentrations (C: <7 at.%, N: <1.1 at.%) and substoichiometric natures (FeO_2.5~2.6_). The PEALD films presented a lower surface roughness (~0.2 nm) and better crystallinity when FeAMD with the similar partial pressure was provided for a single ALD cycle. A major advantage of FeAMD over other Fe ALD precursors identified in this study is that it enables the growth of FeO_x_ films using both H_2_O and O_2_ plasma as co-reactants with practical GPCs in the range of 1.6–1.7 Å/cy at a practical temperature (200 °C). However, the limited thermal stability and low vapor pressure of FeAMD are common to both processes. This problem could potentially be addressed by an advanced reactor design that enables efficient precursor delivery [45]. Despite this limitation, the formation of iron oxide films in the hematite phase with a subnanometer-scale roughness, demonstrated in this study, indicates the possibility of integrating ALD processes with various applications of iron oxides in the hematite phase.

## Figures and Tables

**Table 1:** Comparison of the chemical compositions of the thermal ALD, PEALD, and CVD-like films.

		Atomic Concentrations (at.%)					Atomic Ratio
		C 1s	N 1s	Fe 2p	O 1s	Si 2p	Fe:O
Thermal ALD (H_2_O dose time)	0.1 s	6.7	1.1	38.8	49.4	4.1	2:2.55
	2 s	6.5	0.9	37.5	47.1	8.0	2:2.51
PEALD		6.1	1.1	38.6	50.8	3.4	2:2.63
CVD-like films		14.5	1.7	33.8	43.5	6.6	2:2.58

## Data Availability

The data presented in this study are available on request from the corresponding author.

## Supplementary Materials

The following supporting information can be downloaded at: https://www.mdpi.com/article/10.3390/nano13121858/s1, Figure S1: An example of the pressure log collected during the FeAMD half-cycle, in which the FeAMD dose was repeated five times. In this log, the partial pressure of FeAMD was recorded as 128 mTorr, Figure S2: Pictures of FeAMD precursor of (a) an as-received state, and (b) an agglomerated state; Figure S3: X-ray reflectivity spectra of (a) thermal ALD and (b) PEALD films; Figure S4: GPC of FeO_x_ films as a function of (a) H_2_O dose time (thermal ALD) and (b) O_2_ plasma time (PEALD). Figure S5: N 1s HRXPS of the thermal ALD, PEALD, and CVD-like films.
