# Supplementary material for: Comparative Study of Thermal and Plasma-Enhanced Atomic Layer Deposition of Iron Oxide Using Bis(N,N′-di-butylacetamidinato)iron(II)
Source: Nanomaterials (Basel). 2023 Jun 14;13(12):1858. doi: 10.3390/nano13121858 (PMC10303090; doi:10.3390/nano13121858)
Supplement: Supplementary file 1 [file nanomaterials-13-01858-s001.zip › nanomaterials-2431007-supplementary.pdf]

Supplementary Materials:

Comparative Study of Thermal and Plasma-Enhanced Atomic Layer Deposition of Iron Oxide Using Bis(*N,N′*-di-butylacetamidinato)iron(II)

Boyun Choi †, Gun-Woo Park †, Jong-Ryul Jeong and Nari Jeon *

Department of Materials Science and Engineering, Chungnam National University, Daejeon 34134, Republic of Korea; bow06123@o.cnu.ac.kr (B.C.); adgv9073@gmail.com (G.-W.P.); jrjeong@cnu.ac.kr (J.-R.J.)

* Correspondence: njeon@cnu.ac.kr

† These authors contributed equally to this work.

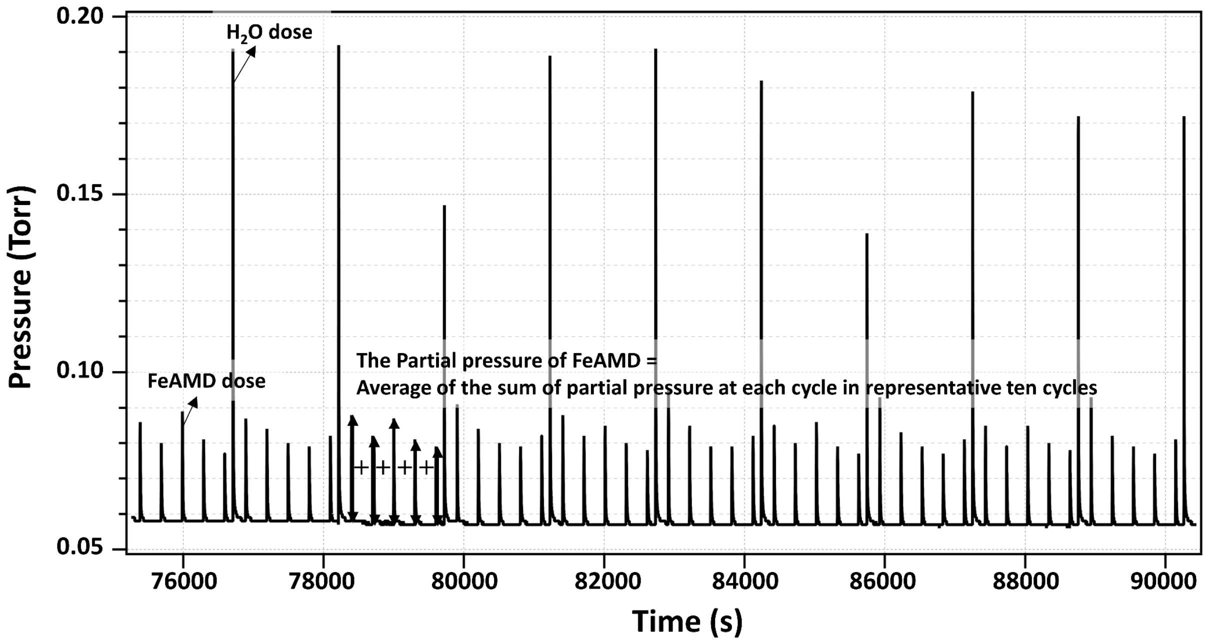

Figure S1. An example of the pressure log collected during the FeAMD half-cycle, in which the FeAMD dose was repeated five times. In this log, the partial pressure of FeAMD was recorded as 128 mTorr.

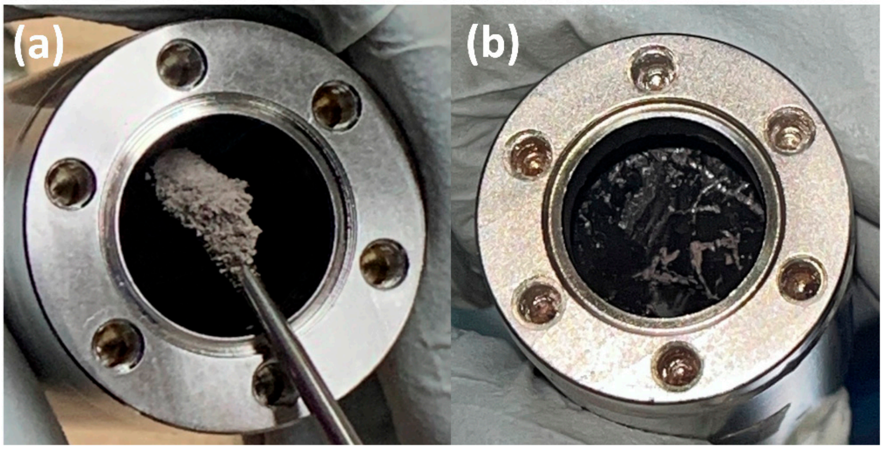

Figure S2. Pictures of FeAMD precursor of (**a**) an as-received state, and (**b**) an agglomerated state.

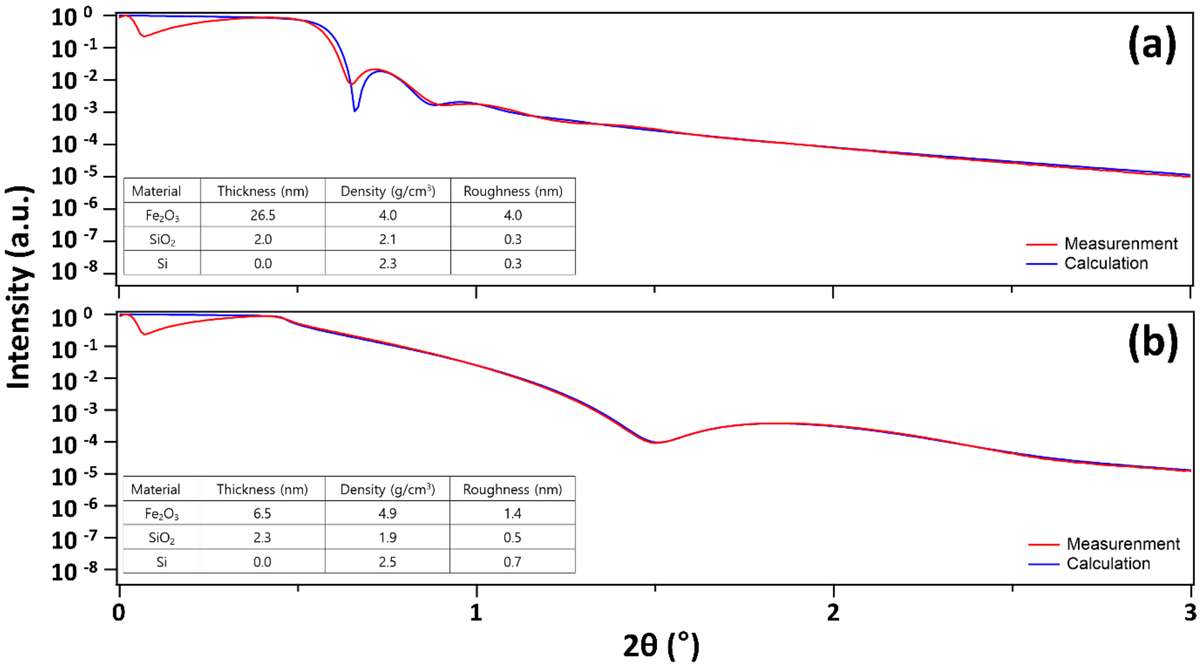

Figure S3. X-ray reflectivity spectra of (**a**) thermal ALD and (**b**) PEALD films.

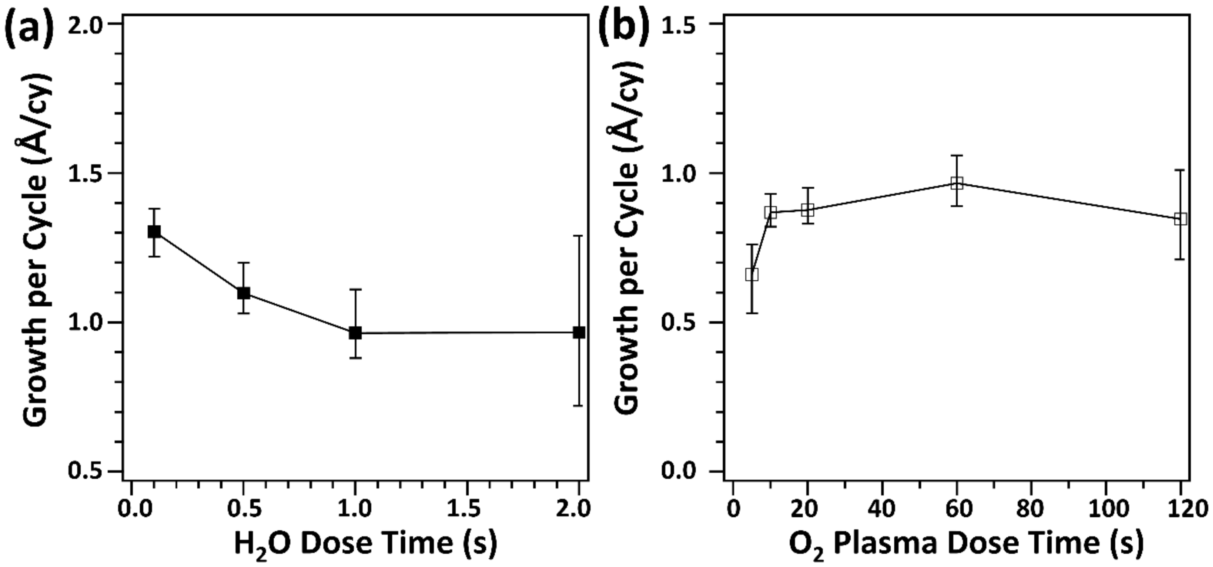

Figure S4. GPC of FeO_x films as a function of (**a**) H_2O dose time (thermal ALD) and (**b**) O_2 plasma time (PEALD).

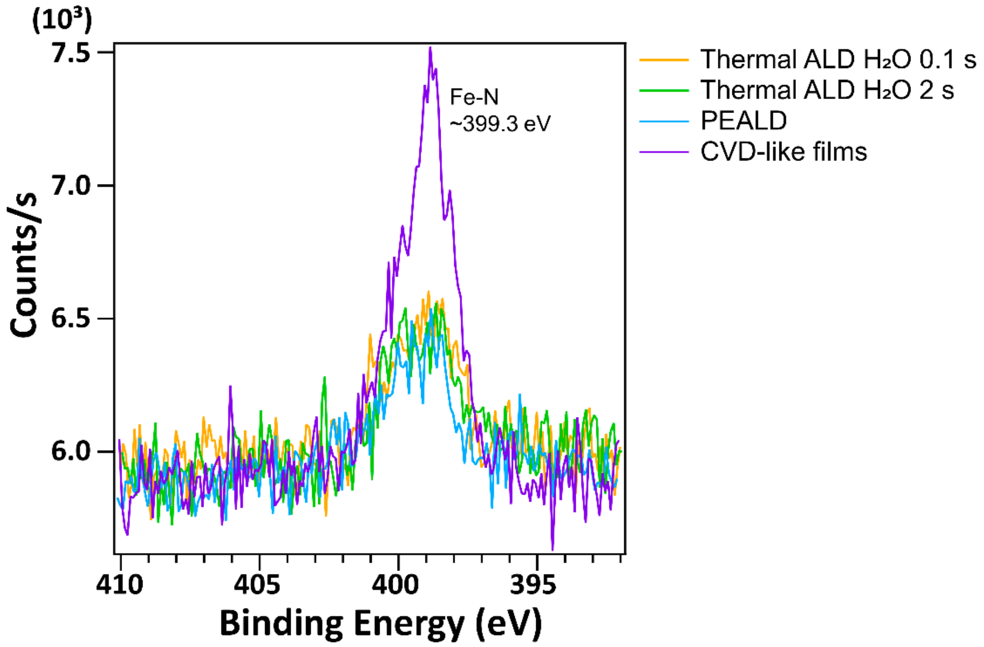

Figure S5. N 1s HRXPS of the thermal ALD, PEALD, and CVD-like films.
